# The Elemental Composition of Demospongiae from the Red Sea, Gulf of Aqaba

**DOI:** 10.1371/journal.pone.0095775

**Published:** 2014-04-23

**Authors:** Boaz Mayzel, Joanna Aizenberg, Micha Ilan

**Affiliations:** 1 Department of Zoology, George S. Wise Faculty of Life Sciences, Tel Aviv University, Tel Aviv, Israel; 2 Department of Chemistry and Chemical Biology, School of Engineering and Applied Sciences, Harvard University, Cambridge, Massachusetts, United States of America; University of Genova, Italy, Italy

## Abstract

Trace elements are vital for the growth and development of all organisms. Little is known about the elemental content and trace metal biology of Red Sea demosponges. This study establishes an initial database of sponge elemental content. It provides the necessary foundation for further research of the mechanisms used by sponges to regulate the uptake, accumulation, and storage of metals. The metal content of 16 common sponge species was determined using ICP measurements. A combination of statistical methods was used to determine the correlations between the metals and detect species with significantly high or low concentrations of these metals. Bioaccumulation factors were calculated to compare sponge metal content to local sediment. *Theonella swinhoei* contained an extremely high concentration of arsenic and barium, much higher (at least 200 times) than all other species and local sediment. *Hyrtios erecta* had significantly higher concentration of Al, Cr, Fe, Mn, Ti and V than all other species. This is due to sediment accumulation and inclusion in the skeleton fibers of this sponge species. *Suberites clavatus* was found to contain significantly higher concentration of Cd, Co, Ni and Zn than all other species and local sediment, indicating active accumulation of these metals. It also has the second highest Fe concentration, but without the comparably high concentrations of Al, Mn and Ti that are evident in *H. erecta* and in local sediment. These differences indicate active uptake and accumulation of Fe in *S. clavatus*, this was also noted in *Niphates rowi*. A significantly higher B concentration was found in *Crella cyatophora* compared to all other species. These results indicate specific roles of trace elements in certain sponge species that deserve further analysis. They also serve as a baseline to monitor the effects of anthropogenic disturbances on Eilat's coral reefs.

## Introduction

The geochemistry of the oceans influences all biological processes and marine life. Of all the elements found in the marine environment, only a dozen are considered “major” elements in biological processes. Most living biomass is chiefly made up of: carbon, hydrogen, oxygen, nitrogen, phosphorus, sodium, potassium, chlorine, calcium, magnesium and sulfur [1], [2]. The proportions of these elements vary within a relatively narrow range in most organisms. In organisms such as demosponges, silicon should also be added to this list. Besides these “major” elements, other elements are found at much smaller or “trace” amounts in all organisms. These elements, particularly first row transition metals such as manganese, iron, nickel, copper, cobalt and zinc, are essential for the growth and development of organisms [1], [2]. While at minute concentrations these trace metals are vital (especially Fe, Co and Zn), they can be toxic at higher concentrations (especially Cu, Pb and Cd) [3]. Due to their importance in the marine environment, trace metals have long been the subject of oceanographic research. Much of this research has focused on the metals uptake by phytoplankton due to their importance as the major primary producers [4]. However, much less is known about the uptake, storage and concentration of trace metals, or their roles in sponge biology.

Scientific interest in the mineral and trace metal composition of sponges began in the 1930's and continued into the 1950's. These works [5], [6], [7] provided our first records of trace metals in sponges and our first insights as to their role and source (also see Noddack 1939, Bergmann 1949, Low 1949 as cited by Bowen and Sutton [6]). However, later research has focused mainly on the suitability and use of sponges as environmental monitors. As stated by Bowen and Sutton [6], in their study of the mineral constituents of sponges, the trace metals found in sponges may come from a number of sources:

1. Sedimentation to which sponges are constantly exposed.
2. Inclusions of sediment and of larger substrate particles by the sponge.
3. Micro-detritus, particles and bacteria filtered by the sponge as food source.
4. Microbial symbionts.
5. Active uptake and accumulation of dissolved trace elements by the sponge.

They also noted the high variability of trace element concentrations measured from specimens within the same species. In some cases a range of concentrations was published, but no statistical analysis was performed [6], [8], [9]. This is due to the relatively large effect of the trace metals content of sediment particles found inside sponges, causing the geological contribution to the measurement to mask the biological one.

When trying to determine the source of the trace metals in sponges one must consider the possibility of species-specific variation. Sponges are active filter feeders, with filtration volumes that may reach between 15000–24000 liters per day per kg sponge [10], [11]. They feed on organic particles, bacteria and even viruses, trapping particles as small as 0.2 microns [11]. Particle uptake and accumulation is affected by variations in clearance rates between species, which depend on the type, size and chemical properties of these particles [12]. Sponges are also affected by differences in mineral preferences [13] and the selective incorporation of foreign particles by different strategies in various sponge species [14]. Dissolved trace metals in the seawater may also be selectively accumulated by sponges [15], [16]. This ability is sometimes dependent on the metals' concentration in seawater [17] while in other cases there is no such correlation [18], [19].

While the Gulf of Aqaba (northern end of the Red Sea) has long been a favored site for coral reef research, relatively little is known about the trace metal content of its sponges. Eilat is located at the northern tip of the long and narrow Gulf of Aqaba. This area is surrounded by arid land and receives little rainfall and negligible water discharge by rivers. Therefore the main external source of trace elements is atmospheric aerosols. These mostly originate from the adjacent lands and are not a result of long-range atmospheric transport [20], [2]. The effect of anthropogenic heavy metal pollution on sediment in this area has previously been studied in Jordan [21] and recently in the Egyptian coast of Sinai [22]. In Eilat itself, monitoring of sediment metal content has been done by the Israeli National Monitoring Program (NMP) and by the Israel Oceanographic & Limnological Research institute (IOLR). These measurements are conducted mainly at sites suspected as polluted, such as ports and marinas, mainly targeting heavy metals and do not include metals of “geological” origin such as Al, Mn and Ti [23], [24]. These factors limit the use of the existing data as a reference for the analysis of trace element content of sponges. The only available data on the elemental content of Red Sea sponges can be found in Pan et al [25]. However, even the latter study is limited to heavy metals and suspected pollutants and was conducted with an emphasis on biomonitoring and not sponge biology.

The current research was aimed at determining the elemental content of Red Sea sponges and gaining a better understanding of the possible roles of trace metals in sponge biology. For this purpose a wide range of metals were measured in various sponge species including on one-hand metals that are usually associated with crustal or geological source (e.g., Al, Mn and Ti), and on the other hand biologically active metals (e.g., Co, Fe and Zn). We hypothesized that a study of the elemental content of 16 common sponge species would result in the elucidation of species-specific differences. Previous research has reported large variation in sponge metal content, possibly a result of sediment content, making standard statistical analyses difficult to perform [6], [26], [8], [9]. We therefore employed a combined use of correlation matrixes and analysis of variation. We assumed that combining these methods and the use of local sediment samples as background references, would enable us to overcome these difficulties. The correlation analysis of a wide range of metals in multiple sponge species can help us understand the possible source of the various metals found in sponges, mechanisms of their accumulation and possibly indicate their biological roles.

## Materials and Methods

The site chosen for this study was in the coral reefs located off the Interuniversity Marine Institute coast of Eilat (Red Sea). This area is presumed to be free of known anthropogenic contribution to the environmental natural metal content. From this area a total of 119 samples of the following 16 sponge species were collected: *Amphimedon chloros, Callyspongia paralia, Callyspongia* sp. (“sticky”), *Crella cyatophora, Diacarnus erythraenus, Haliclona* sp.1 (“tube”), *Haliclona* sp.2 (“blue”), *Hemimycalle arabica, Hyrtios erecta, Negombata magnifica, Niphates rowi*, *Siphonochalina siphonella, Stylissa carteri, Suberites clavatus, Theonella swinhoei*, and *Topsentia aqabaensis*, with at least 3 samples from each species. Specimens were collected according to permits issued by the Israel Nature and National Parks Protection Authority and did not involve any endangered species (permit numbers 2011/38292 and 2012/38779). Sediment samples (n = 3) were also collected from this site.

Sponges were collected by SCUBA diving at depths ranging from 3 m to 30 m. Samples were cut using a ceramic knife to avoid metal contamination, stored in plastic bags or tubes and either processed fresh or kept at −20°C until use. Foreign matter such as macro-detritus, rock fragments and organisms found in the sponge, were removed under a stereoscope using plastic forceps. Sponge samples were lyophilized and 100–200 mg sub-samples were weighed. These sub-samples were digested by boiling in a 5 ml mixture of concentrated 1.25 ml HNO_3_+3.75 ml HCl until a clear solution was achieved and their volume was completed to 20 ml with ddH_2_O.

Elemental content of samples was determined by ICP-AES spectrometry (Inductively Coupled Plasma Atomic Emission Spectrometer) using a Spectro “ARCOS-SOP” (Spectro GMBH, Kleve, Germany) at the Hebrew University of Jerusalem (Faculty of Agriculture, Food and Environment). The elements analyzed were Ag, Al, As, B, Ba, Ca, Cd, Co, Cr, Cu, Fe, Hg, K, Li, Mg, Mn, Mo, Na, Ni, P, Pb, S, Sb, Se, Si, Sn, Sr, Ti, V and Zn. Measurements were calibrated using standard solutions of all tested elements and blanks (Merck standards for ICP). Calibration was continuously verified by standards measurement every 10 samples. The acid solution used for sponge digestion was also used as a control in measurements.

Measurements below calibration or detection values were not included in the analysis and were set as zero. Samples with measurements higher than the calibration values were diluted and measured again. When this was not possible, the highest calibrated value for this element was used. The dilutions were monitored by addition of standards and by monitoring the diluted concentrations of Ca, K, Na, Mg and Sr; elements found in high concentrations in all samples and therefore used as internal standards for dilutions. Background values from the acid mixture and blanks were subtracted from the measurements and values were adjusted using standards. Elemental concentrations were calculated for each sample using its solution volume and the original sample's dry weight (results are shown as mg/Kg). The resulting data set was analyzed using Primer statistical software (Primer 6 with PERMANOVA+ package from Primer-E Ltd.). The results for each element were standardized and a Bray-Curtis resemblance matrix containing the distances or similarities between all data points was produced from all the data. From this a multidimensional projection of all samples (according to their elemental content) was generated using Principal Coordinates analysis (PCO). The various elements were grouped into PCO vectors according to their correlation values (Pearson's coefficient). The PCO vectors' Eigen-values were calculated and vectors explaining at least 1% of the total variation were selected for further analysis. For elements found to be correlated to more than one PCO vector, both the strength of the correlation (R values) and the Eigen-value of the vectors, were considered in the choice of vector. Normal distribution of the selected PCO vector scores was assured by Lilliefors & Shapiro-Wilk tests conducted using Statistica software. Significance of differences between the metal content of the various species was analyzed for each of the selected PCO vector scores by One-Way ANOVA, with “Species” as independent variable. These were followed by Tukey HSD post-hoc tests with p<0.05 considered a significant difference.

The effect of sediment on the sponges' metal content was determined in a separate two-phase analysis. First, a new PCO projection (PCO^II^) was generated based on all sponge and sediment samples. The grouping of elements into vectors according to correlations was compared to that of the previous “sponges only” projection. Only PCO vectors with good correlation to metals and Eigen-value of more than 1% were subject to further analyses. Selected PCO vector scores were further analyzed using ANOVA tests. Following this, a third PCO projection (PCO^III^) was generated based only on the metals selected by the results of the previous “sponge+sediment” (PCO^II^) analysis. It only included those metals that were highly correlated with the sediment samples. The resulting sponges' vector scores of this PCO^III^ analysis were evaluated using ANOVA, as previously described, to test for significant differences between the various sponge species.

The original ICP measurement data set of all sponge elemental values was used to calculate Bio-Concentration Factors (BCF) for all sponge species in this study. The values of elements measured in sediment samples taken from the sponges' immediate vicinity were used as the environmental references. Every sponge elemental measurement was divided by the corresponding elemental value measured in the sediment samples (‘mg/Kg sponge’ divided by ‘mg/Kg sediment’). The resulting ratios showed which elements were concentrated above local environmental values and by which sponge species.

## Results

### Data set

A data set was established for the elements content in the studied sponge species and the sediment samples with some exceptions (all elemental concentrations are shown in SOM Table S1). For most samples Ag, Hg, Pb, Sb and Sn concentrations were below detection threshold of the ICP setup or the values obtained were below calibration limits for these metals. Therefore these elements were not used in compiling the elemental content of the sponge species sampled. P and S (which are common in all organic compounds) and Ca, K, Na, Mg and Sr (which are found in very high concentrations in seawater) were also excluded. The Mo and Se measurements for some of the species were unreliable due to high internal variation or were below calibration and were therefore set as minimal (high variation) or zero (below calibration) for PCO analysis. Mo and Se values for sediment samples were below calibration/detection values, therefore for PCO statistical analysis these values were set as zero. For the calculation of Bio-concentration factors the lowest limits of detection of the ICP setup for these elements were used.

### PCO analysis

**Figure 1:**
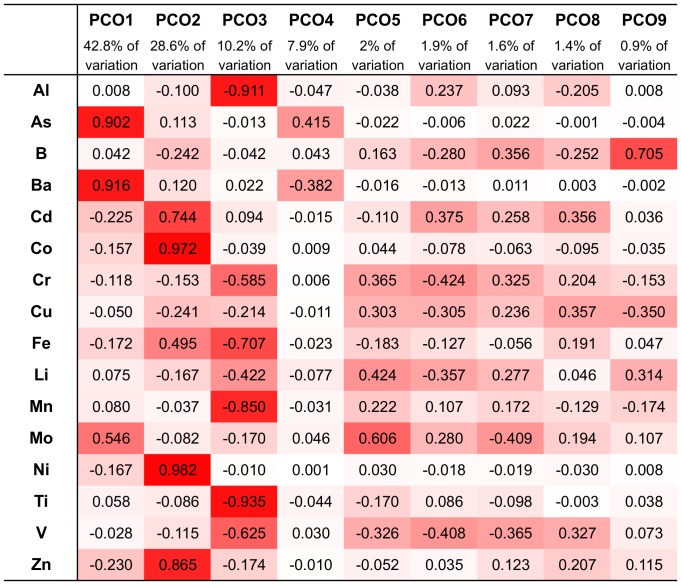
Sponge samples elemental correlation with PCO^I^ vectors (R values), red highlight marks high correlation.

Vectors were created on the basis of correlations between the variables (metals) in the multivariate data set and ranked by their contribution to the total variation between the samples (see Figure 1). Each vector represents a number of metals thereby reducing the number of dimensions needed to visualize the data cloud, allowing the main patterns to be observed.

- Vector PCO1^I^ mainly As and Ba (R>0.9) with partial contribution of Mo (R = 0.54).
- Vector PCO2^I^ mainly of Cd, Co, Ni and Zn (all R>0.7) and partially of Fe (R = 0.5).
- Vector PCO3^I^ mainly of Al, Fe, Mn and Ti (R>0.7) and smaller contributions by Cr (R = 0.58) and V (R = 0.6).
- Vector PCO4^I^ composed of partial contributions of various elements, the highest being As (R = 0.41) and Ba (R = 0.38), all at low correlations.
- Vector PCO5^I^ mainly of Mo (R = 0.6) and a partial contribution of V (R = 0.42).
- Vector PCO6^I^ composed of small contributions by various elements, Cr and V the highest with R>0.4.
- Vector PCO7^I^ composed of small contributions by various elements, Mo the highest with R = 0.4.
- Vector PCO8^I^ composed of small contributions by various elements, all with R<0.4.
- Vector PCO9^I^ composed mainly of Boron (R = 0.7).

**Figure 2:**
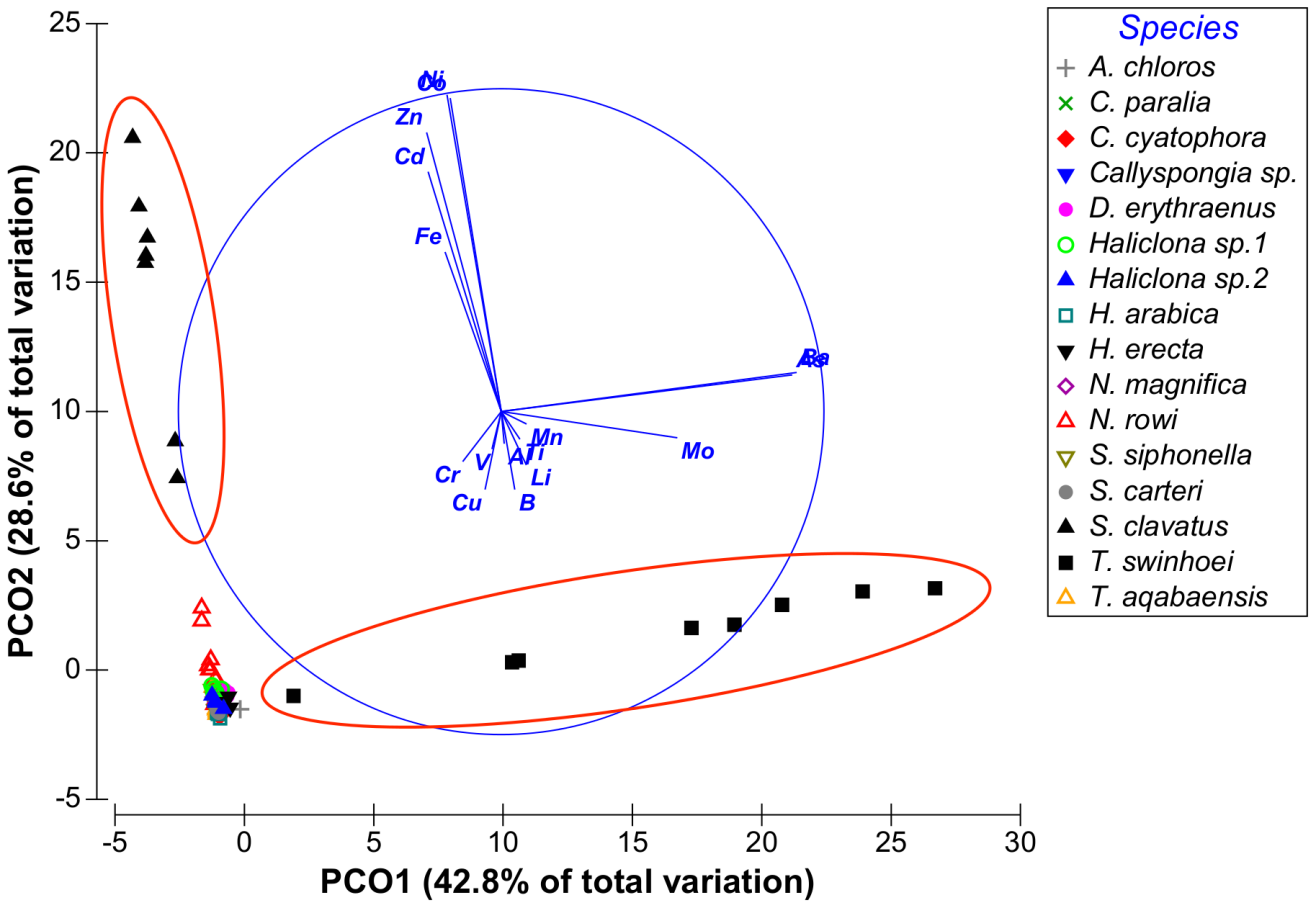
Distribution of sponge samples along PCO^I^ vectors 1 and 2. Species of interest are circled in red.

**Figure 3:**
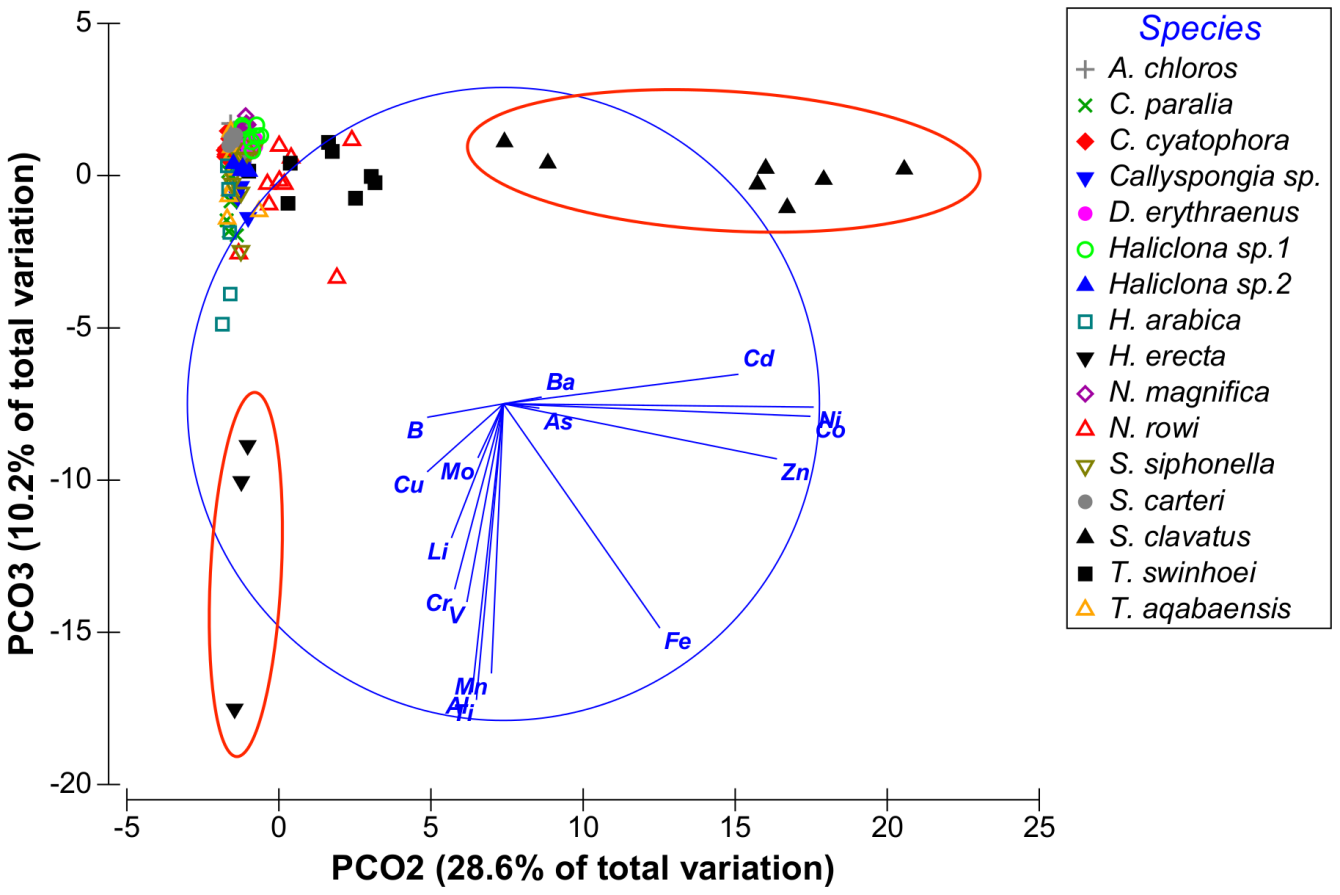
Distribution of sponge samples along PCO^I^ vectors 2 and 3. Species of interest are circled in red.

**Figure 4:**
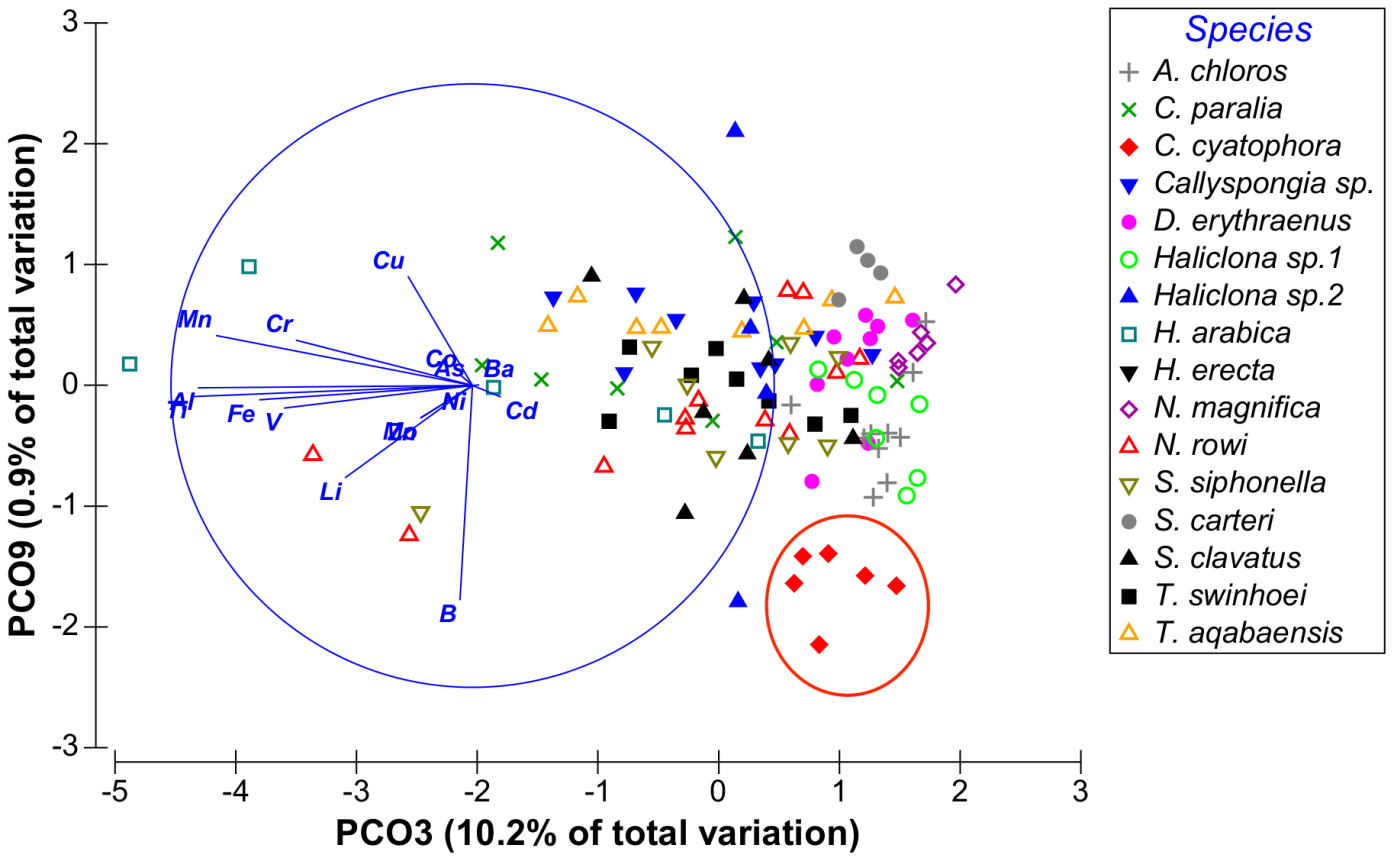
Distribution of sponge samples along PCO^I^ vectors 3 and 9. Species of interest is circled in red.

Although vector PCO9^I^ had only 0.9% Eigen-value it was retained since it is the only vector to show high correlation with B. From these results it was evident that the vectors of interest were PCO1-PCO3^I^, PCO5^I^ and PCO9^I^ having at least one metal correlated with each vector with a correlation value higher than 0.5. Vectors PCO4^I^, PCO7^I^ and PCO8^I^ were not correlated with any of the metals at values higher than R = 0.5 and were therefore not further analyzed. Some metals were found to have partial correlation with more than one vector: Fe was correlated with both vectors PCO2^I^ and PCO3^I^ and Mo was correlated with both vectors PCO1^I^ and PCO 5^I^. The distribution of sponge species according to the main PCO^I^ vectors (vectors 1, 2 and 3) can be seen on Figure 2 and Figure 3. Figure 4 shows the effect of PCO9^I^, the only vector correlated with B, on sponge species grouping.

**Figure 5:**
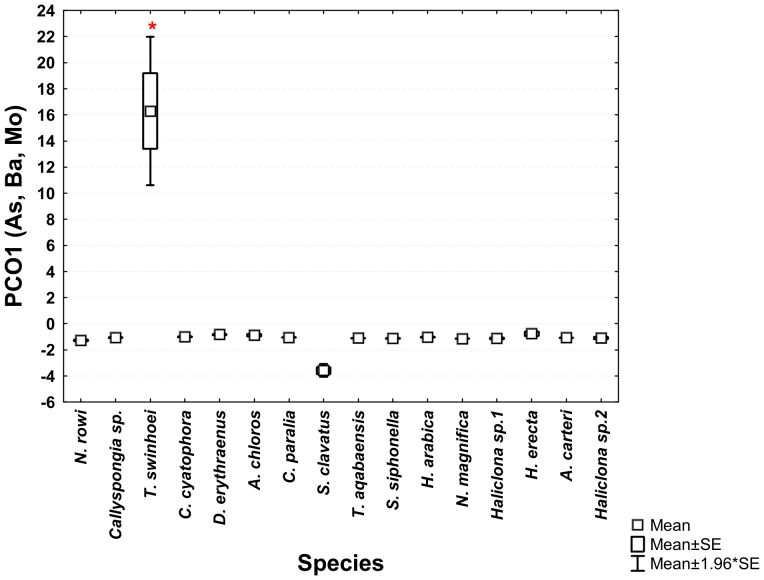
One-way ANOVA of PCO1^I^ vector scores of all studied Red Sea sponge species. Significant results (p<0.05) are marked with a red star.

**Figure 6:**
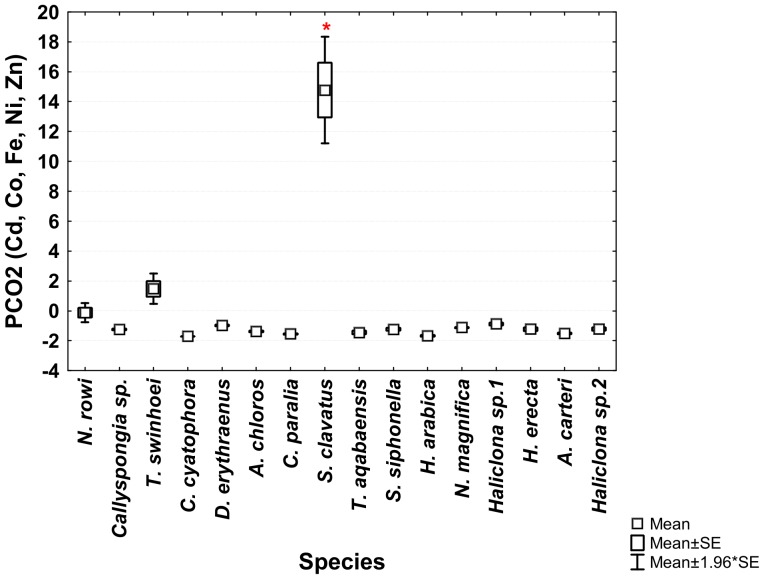
One-way ANOVA of PCO2^I^ vector scores of all studied Red Sea sponge species. Significant results (p<0.05) are marked with a red star.

**Figure 7:**
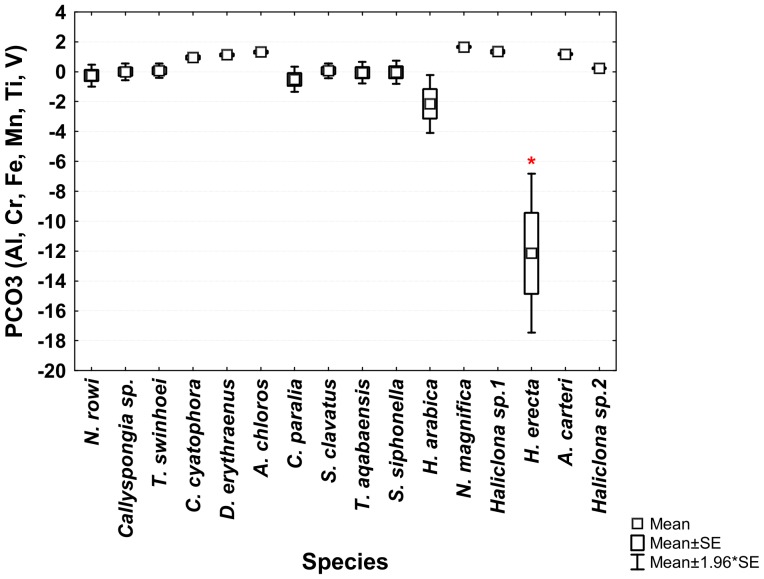
One-way ANOVA of PCO3^I^ vector scores of all studied Red Sea sponge species. Significant results (p<0.05) are marked with a red star.

**Figure 8:**
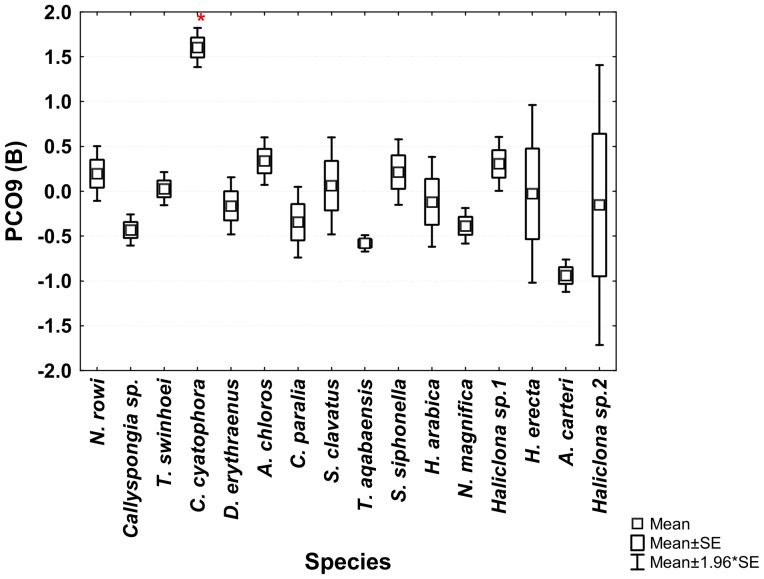
One-way ANOVA of PCO9^I^ vector scores of all studied Red Sea sponge species. Significant results (p<0.05) are marked with a red star.

The ANOVA analysis of the sponges' PCO^I^ scores revealed significant differences between the various species. *Theonella swinhoei* was significantly higher on vector PCO1^I^ (ANOVA, F_1,99_ = 4.7889, p = 0.000147, Tukey HSD test p<0.05) than all other species. This indicates that *T. swinhoei* contains significantly higher amounts of As and Ba than all other species (Figure 5). *Suberites clavatus* scored significantly higher on vector PCO2^I^ (ANOVA, F_1,99_ = 1.7545, p = 0.000147, Tukey HSD test p<0.05). This points to the significantly higher amounts of Cd, Co, Ni, and Zn in *S. clavatus* than all other species (Figure 6). Further analysis of PCO2^I^ also indicated that *S. clavatus* has high Fe content but this metal's contribution to the sponge's significantly high score on this vector is lower than Cd, Co, Ni and Zn due to its lower correlation with PCO2^I^. *H. erecta* scored significantly higher than other species on vector PCO3^I^ (ANOVA, F_1,99_ = 1.2816, p = 0.000147, Tukey HSD test p<0.05) indicating significantly higher content of Al, Cr, Fe, Li, Mn, Ti and V than in all other species (Figure 7). No species showed a significant difference when tested on PCO4^I^ and PCO5^I^. *Crella cyatophora* scored significantly higher on vector PCO9^I^ (ANOVA, F_1,99_ = 0.2843, p = 0.003566, Tukey HSD test p<0.05) than all other species due to its significantly higher amounts of B (Figure 8).

**Figure 9:**
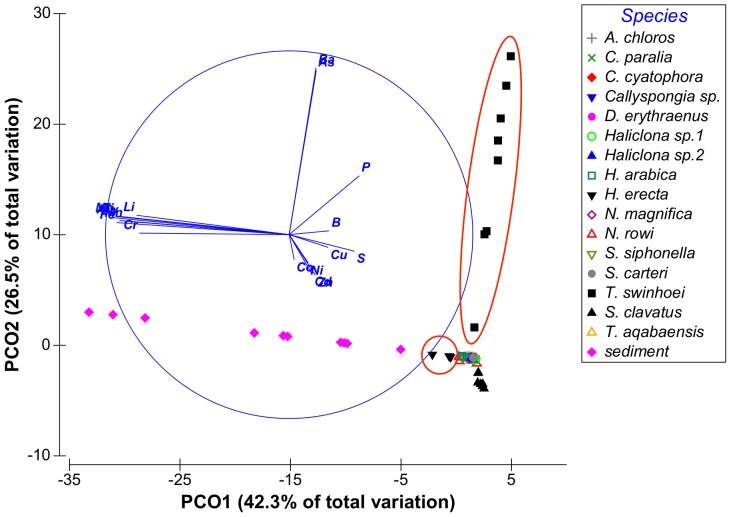
Distribution of sponge and sediment samples along PCO^II^ vectors 1 and 2, explaining 68% of all variation between samples. Samples of interest are circled in red.

**Figure 10:**
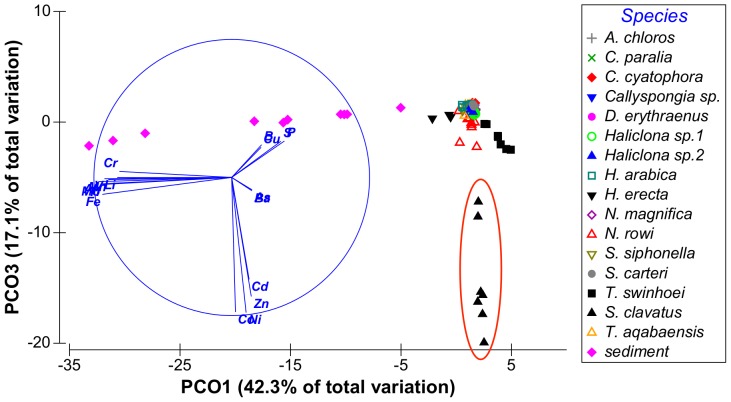
Distribution of sponge and sediment samples along PCO^II^ vectors 1 and 3, explaining 59% of all variation between samples. Samples of interest are circled in red.

To compare the correlations of the metals of interest in the sponges with those of the same metals in the sediment, measurement of sediment samples were added to the previous PCO analysis database and a new correlation matrix was generated. The results of this new PCO vector projection (PCO^II^) showed that vector PCO1^II^ was composed of Al, Cr, Fe, Li, Mn, Mo, Ti, and V which were all highly correlated (R>0.8). Vector PCO2^II^ was mainly composed of As and Ba (R>0.8). Vector PCO3^II^ was comprised of Cd, Co, Ni and Zn, which were highly correlated (R>0.73). Vectors PCO4^II^-PCO8^II^ were composed of small contributions by various elements. Vector PCO9^II^ main contribution came from Cu (R = 0.57) and low contributions (R<0.4) by other metals. Vector PCO10^II^ was composed mainly of a B contribution (R = 0.7) but had a low Eigen value (0.5%). Grouping results for some of the metals differed from the grouping seen by the previous analysis of sponge samples (see SOM Figure S1). The high values of some metals in the sediment skewed the results, affected the correlations and thereby changed the elements' grouping. From these latter results it became evident that the vectors of interest are PCO1-3^II^, which accounted for 85% of the total variation between samples. The distribution of sponge species according to these vectors can be seen on Figures 9 & 10. While most samples are found as a dense overlapping cloud at the center, it is easily noted that all sediment samples are distributed further along vector PCO1^II^. Closer examination of axis PCO1^II^ reveals that *H. erecta* samples are distributed along the same vectors as the sediment samples, further than other sponges but at values much lower than sediment (Figure 9). *T. swinhoei* samples and *S. clavatus* samples are evidently protruding from the main cloud along vectors PCO2^II^ and PCO3^II^ respectively. *N. rowi* follows *S. clavatus* on PCO3^II^ (Figure 10).

**Figure 11:**
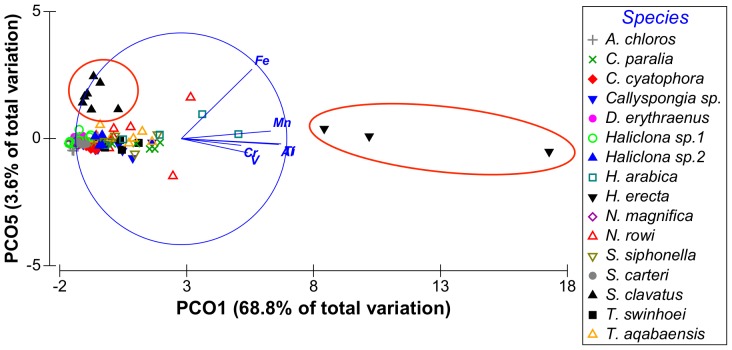
Distribution of sponge samples along PCO^III^ vectors 1 and 5 associated with Fe, explaining 72% of all variation between samples. Species of interest are circled in red.

**Figure 12:**
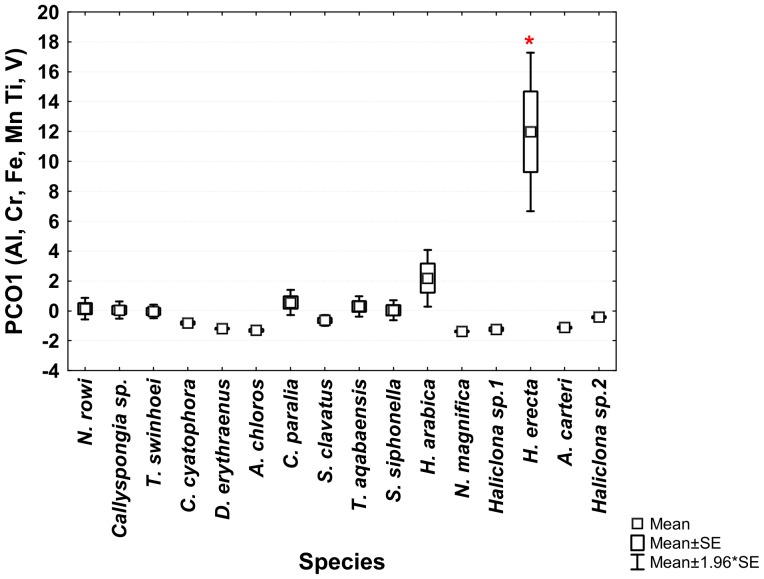
One-way ANOVA of PCO1^III^ vector scores of all studied Red Sea sponges. Significant results (p<0.05) are marked with a red star.

**Figure 13:**
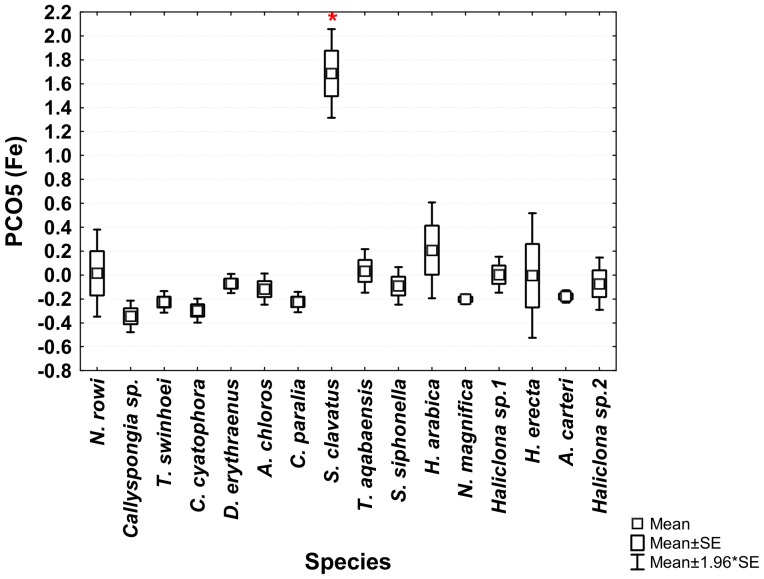
One-way ANOVA of PCO5^III^ vector scores of all studied Red Sea sponge species. Significant results (p<0.05) are marked with a red star.

From the PCO^II^ projection generated by the sponge and sediment data it was determined that Al, Cr, Fe, Mn, Ti and V are all highly correlated and represented by the same vector (PCO1^II^ in Figure 9 and in Figure S1). Sediment samples showed significantly higher scores on this vector (ANOVA, F_1,108_ = 8.23226, p = 0.000159, Tukey HSD test p<0.05) than all other samples (see Figure S2). Therefore a new PCO analysis projection (PCO^III^) was generated using only the Al, Cr, Fe, Mn, Ti and V values of all sponge samples (Figure 11). In this new analysis (see Figure S3) vector PCO1^III^ was composed mainly of Al, Mn and Ti (highly correlated R>0.84) with smaller contributions by Cr, Fe and V (R>0.56). The latter three metals also contributed to the other vectors formed by the analysis. Vector PCO2^III^ was composed mainly of V (R = 0.72) and Cr was the main contributor to vector PCO3^III^ (R = 0.79). Vectors PCO4^III^ and PCO6^III^ were composed of partial contributions from various elements, all at low correlations. Vector PCO5^III^ was composed mainly of Fe (R = 0.63). The PCO^III^ vector scores of all samples were analyzed using One-Way ANOVA for the vectors of interest (1–3 & 5) with “Species” as independent variable and Tukey post-hoc tests were used to determine the significance of the species vector scores. *H. erecta* samples were significantly higher on vector PCO1^III^ (ANOVA, F_1,99_ = 1.2147, p = 0.000147, Tukey HSD test p<0.05) than all sponge species. This reflects the significantly higher amounts of Al, Mn, and Ti and their associated Cr, Fe and V in this species compared to all other sponge species (Figure 12). None of the species scored significantly higher than others on vectors PCO2^III^ and PCO3^III^. *S. clavatus* scored significantly higher on vector PCO5^III^ (ANOVA, F_1,99_ = 0.1067, p = 0.000147, Tukey HSD test p<0.05) than all other species. This result is due to the significantly higher amounts of Fe (with no other associated metals) in *S. clavatus* than all other sponge species and sediment (Figure 13).

### Bio-Concentration Factors

**Figure 14:**
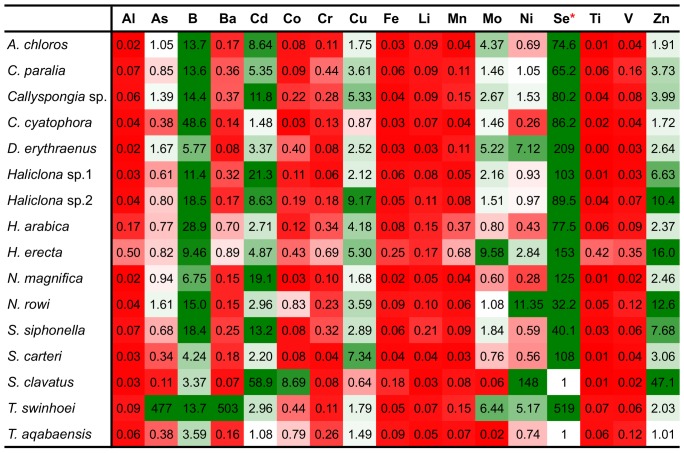
BCFs of all studied metals in Red Sea sponges. Green indicates BCF>1 (darker shades signify higher BCF) and red indicates BCF<1 (darker shades signify lower BCF). * Lowest detection value of ICP setup substituted for missing Se value in sediment, *S. clavatus* and *T. aqabaensis*.

**Figure 15:**
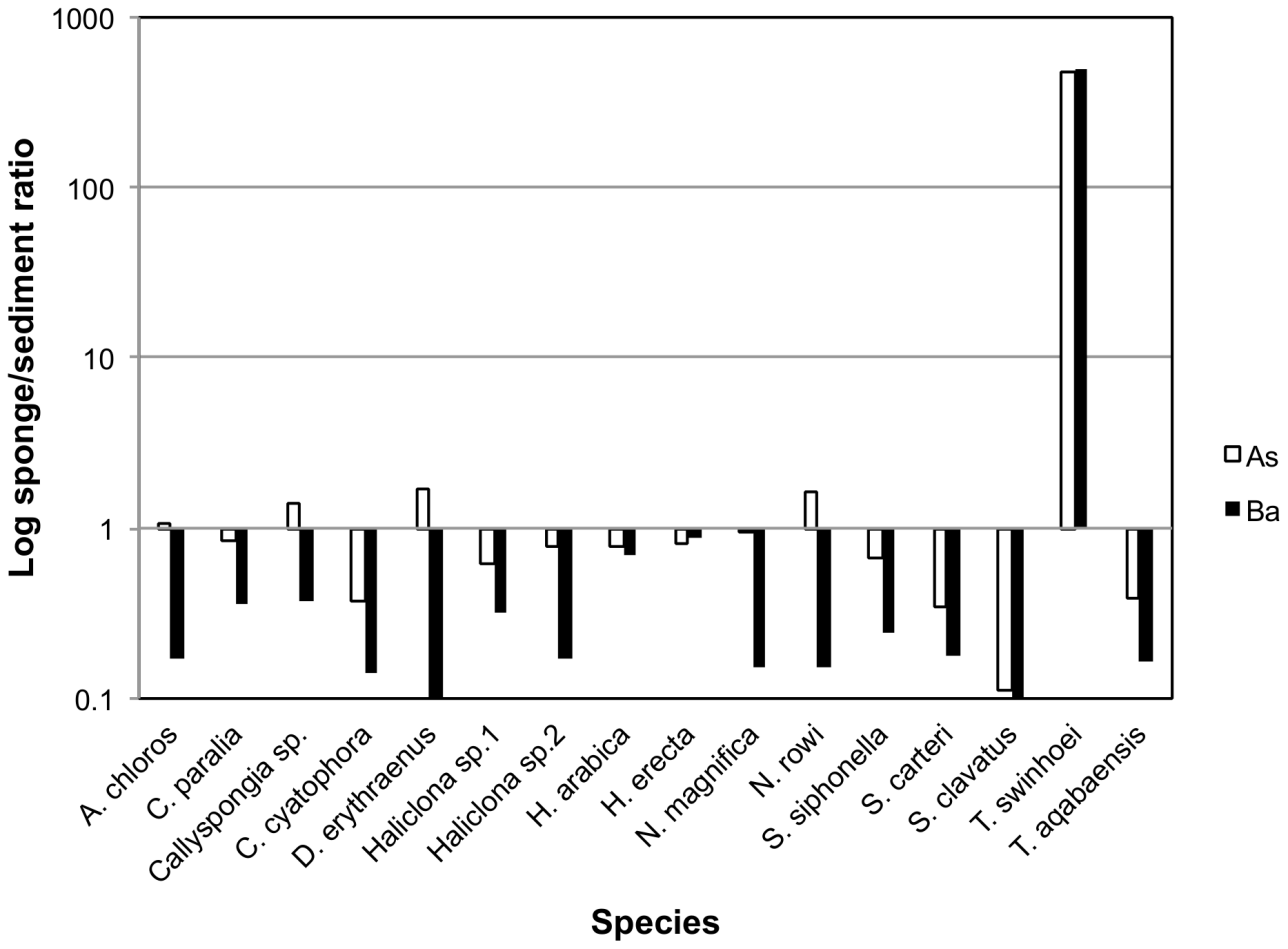
Bioconcentration factors of As (white) and Ba (black) in all sponge species compared to sediment (value set as 1).

**Figure 16:**
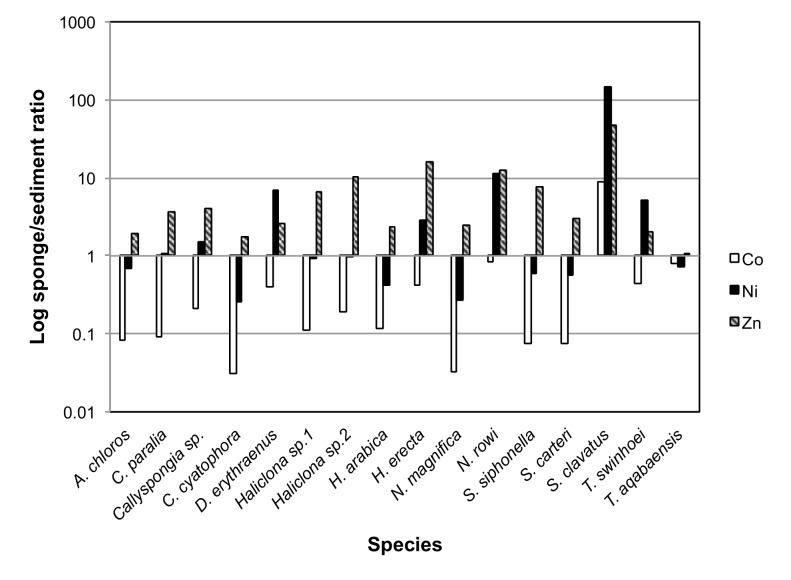
Bioconcentration factors of Co (white), Ni (black) and Zn (grey) in all sponge species compared to sediment (value set as 1).

**Figure 17:**
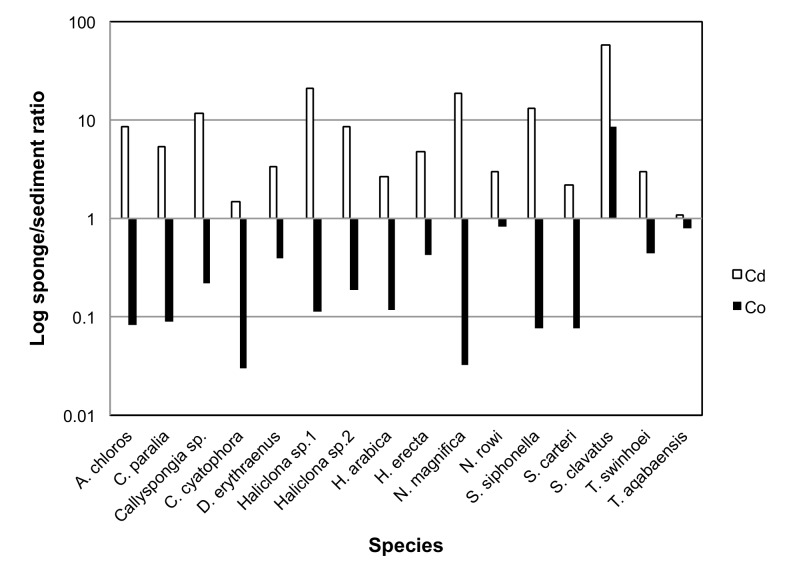
Bioconcentration factors of Cd (white) and Co (black) in all sponge species compared to sediment (value set as 1).

**Figure 18:**
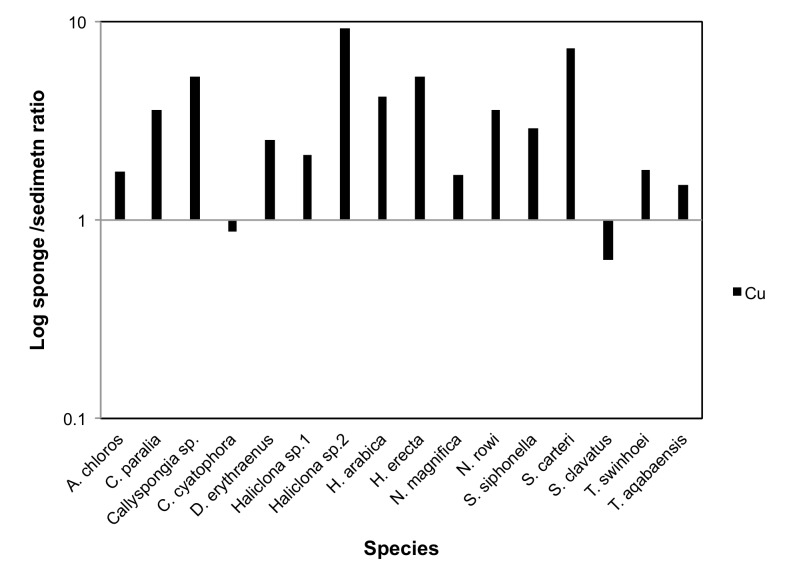
Bioconcentration factor of Cu in all sponge species compared to sediment (value set as 1).

To calculate BCFs (Bio-Concentration Factors), sponge elemental values were compared to those of the same metals measured in the local sediment samples. A BCF value of 1 was set as the benchmark for all elements, with a result of 1 or above being considered indicative of bioaccumulation. The resulting ratios showed that the metals Al, Cr, Fe, Li, Mn, Ti and V were found at much higher concentrations in the sediment than in all sponge species measured. On the other hand the metals B, Cd, Se and Zn were found at higher than sediment concentrations in all sponge species (Figure 14). Se values measured in sediment samples were below detection or below calibrated range and therefore the lowest Se detection value of the ICP setup was used to calculate the BCF for Se. This makes even the low Se values found in almost all sponges highly significant. The only exceptions were *S. clavatus* and *T. aqabaensis* in which Se concentrations were similar to sediment. *T. swinhoei* was found to contain As at concentrations much higher than the sediment (BCF of 477 compared to 1.67 of the next highest species). It is also the only sponge species with Ba higher than sediment with a very high BCF of 503 (Figure 15). The Ni and Zn concentrations found in *S. clavatus* are also much higher than sediment values for these metals (BCF of 148 and 47 respectively). Its BCF for Ni is more that 10 times higher than the next highest species *N. rowi* (Figure 14 and Figure 16). *S. clavatus* is also the only sponge with Co higher than sediment and has a relatively high Cd content as well (Figure 17). *N. rowi* and *T. aqabaensis* are the only other species with a Co concentration approaching sediment values (BCF of 0.83 and 0.79 respectively). *S. clavatus* and *C. cyatophora* are the only species with lower than sediment Cu concentrations (Figure 18).

## Discussion

This research is an analysis of the trace metal content of 16 common Red Sea sponge species. While the trace metal values recorded may serve as baseline value for environmental monitoring, the present study focused on the role of trace elements in Red Sea sponge biology. To this end, the trace metal values measured in the sponges were compared not just between species, but also to measurements of local sediment and to published concentrations of trace metals in Red Sea seawater and sponges. The bio-concentration factors calculated for each metal showed a few interesting trends in the metal content of all the species in this study.

**Boron** is an abundant element in rocks, soil, and water, its average concentration ranging from 100 mg/kg in rocks to about 4.4 mg/kg in seawater [27]. B in the form of borate activates the MAPK pathway, stimulating cell growth and cell proliferation in animals but little else is known about its role in animal physiology. Boron transporters were identified in both plants (BOR1) and mammals (NaBCl) [28]. In this study, B was found to be actively accumulated by all sponge species sampled (Figure 14), demonstrated by their larger than 1 BCFs for this element. According to Furst [29], isotopic evidence suggests a link to silicon uptake kinetics and demand by sponges and that boron and silicon can be discriminated by sponges. Furst stated that demosponge spicules contain relatively large amounts of B, ranging from 500 ppm to 700 ppm [29]. The results shown here for sponge samples are of much lower B concentrations ranging from 10 to 279 ppm. However these measurements are of entire sponge body samples and not of clean spicules, which could result in lower B concentrations. These low concentrations could also be due to the fact that the sponge samples in this study are from warm, low-productivity marine locations whose spicules, according to Furst [29], contain markedly less boron than spicules from temperate, high-productivity regions. Thus the spicules may act as B “sinks”; containing much higher concentration than in the complete sponge body. The spicules' B content would also explain its bioaccumulation by all sponge species in this study.

**Cadmium** in the marine environment has mainly been monitored as pollutant in coastal water due to its toxic effects on humans. However, Cd is apparently an essential element in sponge biology as demonstrated in this study and others. All 16 Red Sea sponge species examined here accumulated Cd (BCF>1 in Figure 14), as do the species previously studied in Saudi Red Sea sites [25]. Such accumulation was also shown for the Antarctic species *Homaxinella balfourensis*, *Mycale acerata*, *Sphaerotylus antarcticus*, *Rossella* sp., *Tedania* sp. and *Axociella* sp. [30], [31]. Cd uptake in sponges most likely depends on seawater, originating either from uptake of dissolved phases or from filtered food particles [31]. This is supported by the Cd concentration in *H. erecta* and *S. carteri* from Eilat, which is in the same range as samples from Saudi control sites even though Cd concentration in Eilat's local sediment is about five times higher than in Saudi sediment. The uptake and concentration of Cd seems to be a highly species-specific process. Some sponges such as *Halichondria panicea* and *Suberites domuncula,* were reported to accumulate Cd in direct proportion to its environmental levels [32], [17], [33], while others such as *Spongia officinalis* accumulate it without such correlation [34]. While obviously Cd is an essential metal in sponges, its role in sponge biology is yet unknown.

**Copper** is an essential trace element and was demonstrated to be required for sponges normal growth and settlement [35], [36]. The results of the current study support these reports. Cu is accumulated (BCF>1) by all tested Red Sea sponge species except *S. clavatus* and *C. cyatophora* (although also for these species BCF was relatively high at 0.64 and 0.87 respectively). The uptake and accumulation of Cu by sponges is a species-specific process. *Crambe crambe* and *Halichondria panicea* accumulate Cu in proportion to its concentration in seawater [37], [17], while *Chondrosia reniformis* regulates Cu levels, unaffected by its concentration at various sites [38]. This is also seen when comparing the Cu concentrations in *H. erecta* and *S. carteri* from Eilat described in this study, to those previously studied in Saudi Arabia. *H. erecta* from Eilat had Cu concentrations at the same range as samples from all Saudi sites, but with very high variation (22±14 g/Kg). Cu concentrations in *S. carteri* from Eilat are higher than those at all Saudi sites even though Eilat's local sediment Cu concentration is in same range as Saudi control sites. In both *H. erecta* and *S. carteri* from Saudi Arabia it was apparent that Cu concentration is not dependent on its concentration in local sediment [25]. Therefore it is suggested that the Cu content of both these species is a result of active Cu uptake from seawater, while the actual concentration is subject to species-specific regulation. The biological roles ascribed to Cu and its ability to adopt various redox states [39] may account for the high variation in its concentration between the species studied here. This ranged from 2.7 g/Kg for *S. clavatus* to 38.6 g/Kg for *Haliclona* sp.2, a range comparable to values recorded from species all over the world [9], [40], [17], [41].

**Selenium** natural concentrations are very low, with 0.05–0.09 ppm in the earth's crust and usually only 0.45 ppb in seawater [27]. Therefore it was surprising to discover that all sponge species in this study accumulated Se when compared to local sediment samples (BCF>1, Figure 14). The only exceptions are *S. clavatus* and *T. aqabaensis* for which Se values were very low, below calibration values, as were the sediment samples. Thus, detection of even these lowest Se concentrations in the sponges is highly significant and indicate active uptake of Se by sponges. One explanation for this Se selection is its involvement in demosponge spicule-genesis proteins and in selenoprotein M, as has been found in *S. domuncula* [42], [43]. Such activities could be present at different levels in all sponge species. However the low Se concentration in *S. clavatus*, the species with the highest spicule content in this study, does not support this explanation. Another source of Se in sponges could be their associated bacteria, for which Se is known to be an essential trace element. Bacteria incorporate Se into selenocysteine, selenomethionine and selenoenzymes [44]. The highest concentration of Se measured here was in *T. swinhoei*. This species harbors a large amount of symbiotic bacteria, which may lead to its high Se concentration (values range 15–38 ppm, more than twice the amount found in all other sponge species). Further experiments might determine whether Se is concentrated in *T. swinhoei* sponge cells or in its associated bacteria.

**Zinc** is an essential trace element in plants, animals and microorganisms. It is the second most abundant transition metal in organisms after Fe, and the only metal represented in all six classes of enzymes [45]. Therefore, it is not surprising that Zinc is actively accumulated by all sponge species in this study as reflected by their larger than 1 BCF for Zn (Figure 14). The sources of Zn uptake by marine sponges are unknown. It was suggested to originate from dissolved Zn in seawater, the same as Si which has a similar oceanic distribution pattern [46], [41], [26]. This was also implied in a work on *Halichondria panicea* [17]. An alternative proposal was that the main Zn source of uptake is particulate organic matter (POM) ingested as food by the sponge and not the dissolved Zn in seawater [47], [48]. A comparison of Zn concentration in the sponges *H. erecta* and *S. carteri* in this study and in Saudi Red Sea sites [25], also indicates that sponge concentrations are not dependent on the sediment Zn concentrations, but on Zn uptake from seawater. However, further research is necessary to determine whether this originates from dissolved Zn phases or POM.

The analysis of bio-concentration factors showed some metal accumulation trends common to all sponge species and some typical of specific species. All sponges were collected from same location and subject to the same environmental conditions, including sedimentation rates, and to the metal content typical of local geology. This approach results in the same amount of incidental sediment in all samples and all species. Therefore any significant difference found in metal content between various species, must be due to species-specific variation. In the following section the biological significance and potential origin of the trace metals found in the sponge species of interest will be discussed.

***Theonella swinhoei*** was found to contain significantly higher concentration of arsenic and barium than all other species (Figure 5). Their concentration in *T. swinhoei* was at least 100 times that of all other species and at least 400 times higher than that of local sediment samples (Figure 14). Therefore these concentrations are clearly not simply a result of accumulated sediment particles. These findings lead to the conclusion that both As and Ba are actively accumulated by the sponge from its environment.

Arsenic is a naturally occurring toxic metal commonly used in the past as pesticide. In coastal regions, As^5+^ is usually found as the predominant form of arsenic species in oxygenated seawater. A study of *Halichondria* sp. from Japan suggested that after its uptake, As is detoxified and stored in the sponge tissue by its conversion to arsenobetaine and arsenosugars [18]. Arsenic acute toxicity decreases with progressive methylation in the following order: As^5+^>As^3+^>methylarsonicacid>arseno-sugar>arsenobetaine. While arsenosugars are found in microalgae that may exist in the sponge as symbionts or as ingested food particles, in most sponges arsenobetaine is the more dominant form of storage. In Japanese *Theonella* sp. arsenobetaine was correlated with the total amount of water-soluble As found in the sponge. Therefore it was suggested that sponge As concentration is more influenced by sponge-made arsenobetaine rather than by symbiont/microalgae arenosugars [19]. The Japanese *Theonella* sp. was also shown to have higher As concentration than other species in Japan (157 mg/kg), but less than a tenth of that shown locally by *T. swinhoei* (Table S1). Whether As is actively uptaken and accumulated by the Red Sea sponge *T. swinhoei* itself or by its many symbionts has yet to be experimentally determined.

Little is known about the role of barium and its metabolism in sponges. Ba^2+^ ions may inhibit the development of dormant *Spongilla lacustris* gemmules [49] and have been reported to act as an AmqKir ion-channel blocker in *Amphimedon queenslandica* [50]. While water-soluble barium is highly toxic, its minerals Barite (BaSO_4_) or Witherite (BaCO_3_) are commonly found in the environment. Barite is mostly found in seawater as micro-crystals mineralized by phytoplankton [51]. The extremely high concentration of Ba in *T. swinhoei*, the only sponge species with Ba concentration higher than the sediment, may be a result of feeding on Barite-producing algae and retention of their Ba content. The very large amount of diverse microbiota hosted by *T. swinhoei* could also be the source of its high Ba concentration. It is also possible that barium is uptaken by the sponge itself in its dissolved toxic form and then metabolized and detoxified as has been suggested for the As detoxification metabolism of some sponge species [18]. The accumulation of Ba and As by either of these sources could be potentially linked and is indicated by their grouping in the PCO analysis. The true source and metabolism of Ba in *T. swinhoei* is yet to be determined.

***Hyrtios erecta*** contained significantly higher concentration of Al, Cr, Fe, Mn, Ti and V than all other species (Figure 7). All these elements were grouped together into vector PCO3 by the analysis (Figure 2). Al, Mn and Ti were highly correlated with vector PCO3 while Cr, Fe and V were at a lower correlation (The latter elements were also partially correlated with other vectors). Although significantly higher than in all other species, the concentration of all these elements in *H. erecta* was lower than found in the local sediment (Figure 14). This called for a closer look at each of these elements and their involvement in sponge metabolism.

*H. erecta* belongs to the order Dictyoceratida, family Thorectidae [52]. Species within this family lack spicules and have a highly developed fiber skeleton. Both primary and secondary fibers can be heavily cored by foreign material and therefore the sponge accumulates a large amount of detritus [52]. The inclusion of sediment particles by *H. erecta* would therefore result in high concentration of the metals present in the local sediment in large amounts. This is in accordance to the PCO analysis of all sponge samples and local sediment samples. Indeed Al, Mn, Ti as well as Cr, Fe and V are all correlated and grouped into the same vector in both sediment and *H. erecta* (Figures 7 & 8). Regrettably no data on Al, Mn and Ti sediment content has been published for Eilat in recent years. The two works published on Red Sea corals and sponges as biomonitors also lack measurements of Al, Fe, Mn and Ti needed for comparison to the current data and the analysis of sediment contribution to the sponges' elemental content [53], [25]. However, the same correlations of Fe/Ti, Fe/Cr and Fe/Mn were noted by Araujo et al. [26] in their analysis of sediment samples. A study of dry deposition of dissolved trace metals in the Gulf of Aqaba also grouped Al, Fe and Mn together as having crustal or geological origin [3]. The conclusion therefore is that the high Al, Cr, Fe, Mn, Ti and V concentrations found in *H. erecta* are a result of sediment accumulation and inclusion in its skeleton fibers and not due to active uptake of these dissolved elements from seawater.

***Suberites clavatus*** contained significantly higher concentration of the elements Cd, Co, Ni and Zn than all other species (Figure 5). All these elements were grouped together with high correlation by the PCO vector analysis (Figure 2). It also had a relatively high Fe concentration, the second highest after *H. erecta*. The use of Al as a proxy for crustal or geological origin of elements helps us determine the source of Fe. While *H. erecta* has a nearly 1∶1 ratio of Al and Fe, *S. clavatus* exhibits a low Al/Fe ratio (also found in *N. rowi*). This is seen in the PCO^III^ analysis projection of all sponge species generated using only the concentrations of the elements (Al, Cr, Fe, Mn, Ti and V) previously grouped as having “geological” origin (Figure 11). The fact that *H. erecta* is higher on an axis grouping Al, Mn and Ti with an associated lower Cr, Fe and V contribution while *S. clavatus* scored significantly higher on the “clean” Fe axis attests that in the latter species “geological” Fe has a relatively small contribution to the Fe content. This Fe origin is surprising considering the numerous sediment inclusions found in *S. clavatus* [54]. It is suggested that this phenomenon reflects a selective intake or selective retention of sediment particles resulting in accumulation of Fe without its geologically associated elements. Such mineral sensitive mechanisms have been previously shown in the sponges *Chondrosia reniformis* and *Dysidea etheria* [13], [14]. An alternative possibility is the active uptake of dissolved Fe by metal ion transporters such as siderophores or metal-binding cyclic peptides [55]. Thus the exact mechanism of Fe accumulation in *S. clavatus* (and perhaps in *N. rowi*) still needs to be experimentally determined.

*S. clavatus* also contained significantly higher Cd concentration than all other species in this study (Figure 6). High Cd concentrations have been shown to cause a reduction in filtration rate in *H. panicea* [32], interfered with cell regulatory processes and disrupted aggregation responses of *Microciona* cells [56]. High Cd levels may also trigger apoptosis in *S. domuncula* [57]. However, some species such as *Tedania charcoti* accumulate extraordinarily high Cd levels (2000–15000 ppm) [15]. *S. clavatus* Cd concentration is over 50 times higher than recorded in local sediment samples −0.09 ppm. This value indicates that Cd is active uptaken and accumulated from seawater, in which values are as low as 0.008–0.013 ppb [23]. Such accumulation could be achieved by using the same ion channels and transport systems as Ca^2+^, as suggested by Phlip [58].

*S. clavatus* also contained Co at significantly higher concentration (nearly 10 times) than all species in this study (Figure 6). It is the only Red Sea sponge species with Co concentration higher (more than 8 times) than the local sediment (Figure 14; Figure 17). These results show that Co uptake in *S. clavatus* is an active process. Whereas Cobalt is a known cofactor for specific enzymes in diatoms and cynobacteria [59], its role in sponge metabolism is virtually unknown. Co concentration in Eilat seawater is in the range of 0.025–0.038 ppb [23]. Co uptake is perhaps mediated by high and preferential permeability of the sponge cell membrane, or even via some form of active transport system such as previously suggested for *Spirastrella cuspidifera* [60].

*S. clavatus* Ni concentration range was 267–781 ppm. Even at its lowest, it is significantly higher than all other species. This cannot be attributed to the uptake of sediment particles with high Ni content since *S. clavatus* BCF for Ni is more than 100 (Figure 16). Exceptionally high Ni levels (2400–4300 ppm) were measured in *Suberites carnosus* in Portugal, which also were not explained by environmental Ni levels [26]. The uptake and accumulation of Ni by *S. clavatus* is therefore an active process that may also occur in other members of the *Suberites* family. This process could be mediated by proteins similar to those isolated from *Cliona viridis* [61].

Finally, *S. clavatus* exhibited significantly higher Zn content (563–1146 ppm) than all other species studied here (Figure 6) with a BCF of 47 compared to local sediment, nearly 10 times that of other species (Figure 16). This level is similar to the congeneric *S. carnosus* from Madeira (460–550 ppm). For other sponges with very high Zn content (e.g., *Tedania charcoti*, 5100–5000 ppm and *C. viridis*, 4700–6700 ppm) it was suggested that Zn may either have an antibacterial and antifouling role [15], or that it is the end product of detoxification system in the form of zinc phosphate granules [8], respectively. However, such granules were not detected in *S. clavatus* microscopical examination. An alternative explanation for Zn role in *S. clavatus* is hypothesized here. Among all tested Red Sea sponge species *S. clavatus* has the highest percentage of inorganic content and the highest spicule content [62]. Therefore, the high Zn content found in *S. clavatus* may be incorporated within its spicules as has been previously shown in hexactinellid sponges [47], [48]. This explanation would be in agreement with Mediterranean sponge data, which show *Cinachyrella levatinensis* as having both high Zn concentrations and high spicule content [63], [64]. This idea is further supported by findings that Zn concentration in *Halichondria panicea* increased upon exposure to higher seawater Zn concentrations and did not decrease even when seawater Zn concentration declined [17].

***Crella cyatophora*** had a significantly higher B concentration (values range 180–279 ppm) than all other species (Figure 8). Since demosponge spicules contain relatively large amounts of B [29], sponges with high spicule content could have higher B content than species with low spicule content. If presence of B in spicules explains its content in the sponge, we could expect *S. clavatus*, which has the highest spicule content (spicule/sponge wet weight) [62], to have the highest B concentration. However it is *C. cyatophora*, with the second highest (but much lower) spicule content, that had the highest B content, while in this study *S. clavatus* had the lowest B content (15 ppm). Furst [29] has also reported that B is diet-derived since the spicules B content correlated better with the environment from which the food originated than with the environment of the sponge itself. If this is valid, the differences in B content between *C. cyatophora* and *S. clavatus* (both species with high spicule content) could reflect differences in their food intake selection.

### Conclusions

This initial survey of elemental content within northern Red Sea sponges was oriented towards a better understanding of trace metals role in sponge biology. The metals Al, As, B, Ba, Cd, Co, Cr, Cu, Fe, Li, Mn, Mo, Ni, Se, Ti, V and Zn were measured in 16 Red Sea sponge species. The various species and metals were all compared to local sediment samples and to published seawater values, to determine BCFs for all sponges. The results show that all sponges actively accumulate B, Cd, Se and Zn at higher concentrations than exist in the local sediment. Other metals, such as As, Ni and Mo are accumulated only by some species. Another pattern that was noted is that sponges with high Cd concentrations (*Haliclona* sp.1, *N. magnifica* and *S. siphonella*) had a low Co concentration, and those with high Co had relatively low Cd (*N. rowi, S. clavatus* and *T. aqabaensis*). Cd and Co were shown to be interchangeable ions for Zn in diatoms [65] and competitive inhibition of Mn uptake by Cd was demonstrated in phytoplankton [66]. Cd and Co may share the same uptake mechanism or perhaps compete for “storage” within the sponge, thereby resulting in opposing trends.

Principal Coordinates analysis was used to decipher relations and correlations between elements in sponges and sediment by grouping elements into vectors. The sponges' PCO scores were then tested using ANOVA to determine significance of high and low elemental values in species. The concentrations of As, Ba and Se found in *T. swinhoei*, Cd, Co, Fe, Ni and Zn in *S. clavatus*, Al, Cr, Fe, Mn, Ti and V in *H. erecta* and B in *C. cyatophora*, demonstrated sponge species-specific ability to selectively uptake and accumulate these elements from their environment. Based on these results it is suggested that such a statistical “toolkit” of combining PCO analyses with ANOVA gives a better picture than the more commonly use of multiple ANOVA for this type of database. It addresses the problems created by high variation of sponge elemental measurements caused by the inherent nature of sponges as “clogged filters”.

Although this research was not oriented towards biomonitoring, the results could serve as a baseline for the elemental content of 16 coral reef sponge species. These data together with environmental data collected by existing monitoring programs could be used to monitor the effects of anthropogenic disturbances on local northern Red Sea coral reefs. It could also facilitate comparison to coral reef data sets from other Red Sea locations such as the Sinai and Saudi Red Sea coasts [53], [25].

Employing additional new methods and techniques will enable the investigation the various mechanisms employed by sponges and their associated microbiota, to specifically accumulate different metals, and the role of these metals in sponge and its associates biology and metabolism.

## Supporting Information

Figure S1 **Sponge and sediment samples elemental correlation with PCO^II^ vectors (R values).** Red highlight indicates high correlation.
(TIF)

Figure S2 **One-Way ANOVA of PCO1^II^ vector scores of all studied Red Sea samples.** Significant results (p<0.05) are marked with a red star.
(TIF)

Figure S3 **Sponge samples elemental correlation with PCOIII vectors (R values) based on metals previously correlated with sediment.** Red highlight indicates high correlation.
(TIF)

Table S1 **Range of elemental concentrations (mg/Kg) in studied Red Sea sponge species.** Measured range is shown for each element in each species.
(PDF)
