# Correction: The Elemental Composition of Demospongiae from the Red Sea, Gulf of Aqaba

**DOI:** 10.1371/journal.pone.0099918

**Published:** 2014-06-04

**Authors:** The PLOS ONE Staff

## Notice of Republication

This article was republished on May 20th, 2014, due to Figure 16, Figure 17, and Figure 18 being ordered incorrectly. Please download this article again to view the correct version. The originally published, uncorrected article and the republished, corrected article are provided here for reference.

## Supporting Information

File S1 **Originally published, uncorrected article.**
(PDF)

File S2 **Republished, corrected article.**
(PDF)
