# Supplementary figures and images for: The Elemental Composition of Demospongiae from the Red Sea, Gulf of Aqaba
Source: PLoS One. 2014 Apr 23;9(4):e95775. doi: 10.1371/journal.pone.0095775 (PMC3997428; doi:10.1371/journal.pone.0095775)

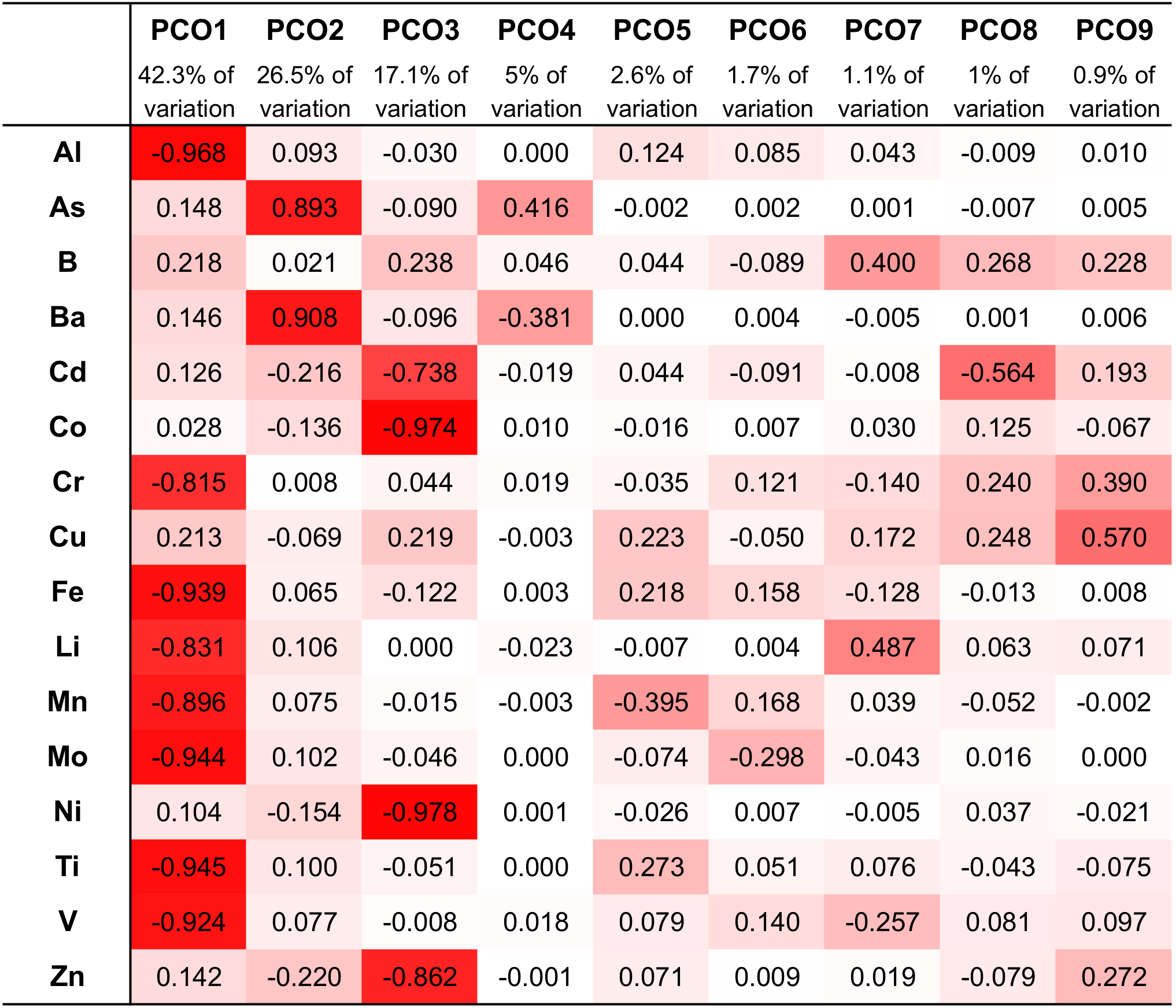

Supplement: Figure S1 — Sponge and sediment samples elemental correlation with PCOII vectors (R values). Red highlight indicates high correlation. (TIF) [file pone.0095775.s001.tif]

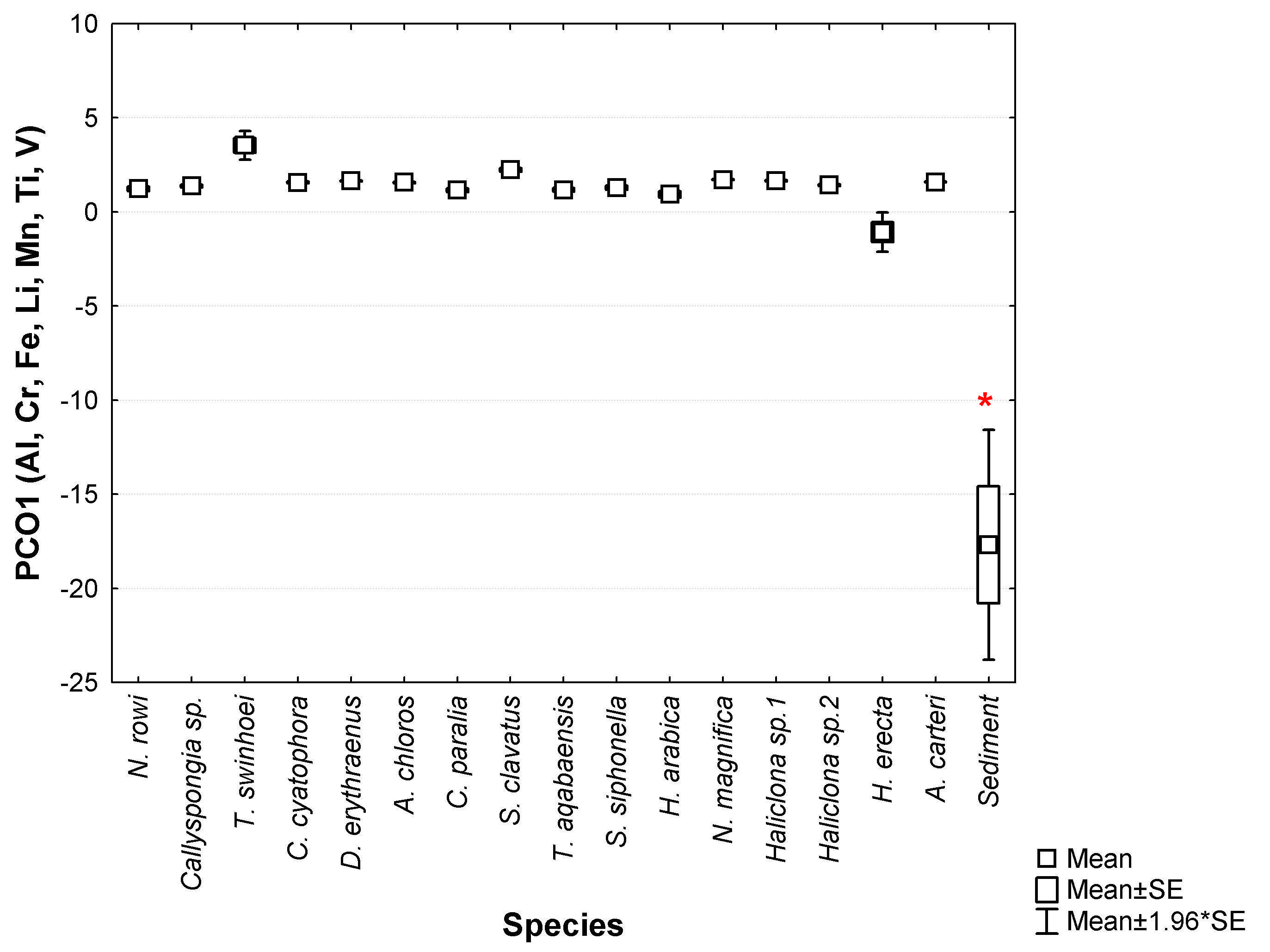

Supplement: Figure S2 — One-Way ANOVA of PCO1II vector scores of all studied Red Sea samples. Significant results (p<0.05) are marked with a red star. (TIF) [file pone.0095775.s002.tif]

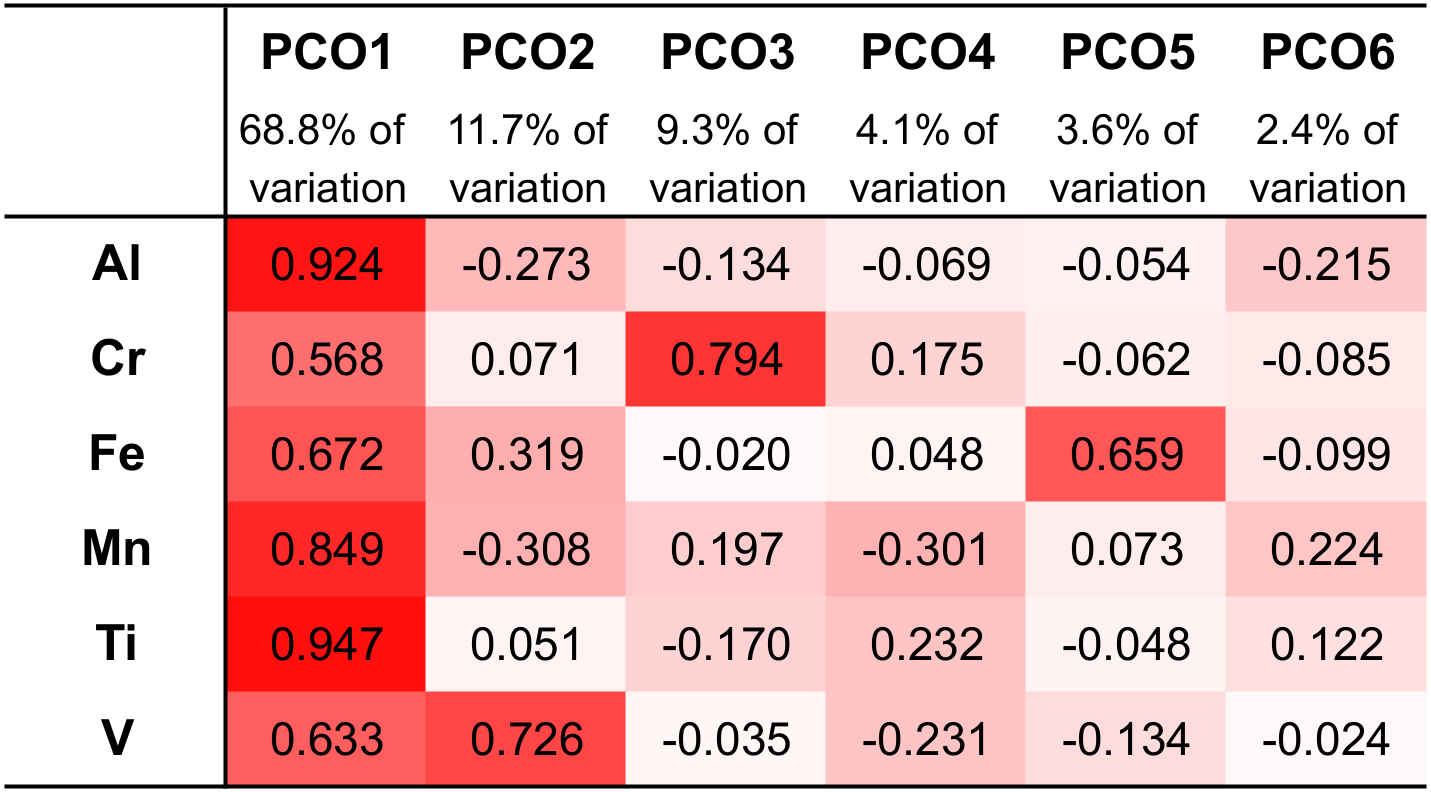

Supplement: Figure S3 — Sponge samples elemental correlation with PCOIII vectors (R values) based on metals previously correlated with sediment. Red highlight indicates high correlation. (TIF) [file pone.0095775.s003.tif]
