# Supplementary material for: The Elemental Composition of Demospongiae from the Red Sea, Gulf of Aqaba
Source: PLoS One. 2014 Apr 23;9(4):e95775. doi: 10.1371/journal.pone.0095775 (PMC3997428; doi:10.1371/journal.pone.0095775)
Supplement: Table S1 — Range of elemental concentrations (mg/Kg) in studied Red Sea sponge species. Measured range is shown for each element in each species. (PDF) [file pone.0095775.s004.pdf]

Species	Al	As	B	Ba	Cd	Co	Cr	Cu	Fe	Li	Mn	Mo	Ni	P	S	Se	Ti	V	Zn
A. chloros	22	8.34	19.0	0.56	0.25	0.16	0.20	2.25	29	0.44	1.33	0.19	1.32	460	7021	0.92	0.91	0.16	11.2
**n=11**	115	42	88	5.95	1.54	0.64	1.64	11.3	797	2.06	9.7	4.91	4.9	3549	39007	6.59	24	2.03	56
C. paralia	41	8	41	0.80	0.20	0.16	0.50	8.81	128	0.51	1.65	0.17	2.69	937	10287	1.12	2.57	0.21	30
**n=8**	305	36	84	5.63	1.11	0.36	6.54	22	660	1.82	16.8	0.66	6	4973	33047	6.45	25	2.93	124
***Callyspongia* sp.**	81	17.1	47	1.45	0.52	0.48	0.43	13.1	90	0.27	5.11	0.53	4.05	1861	22163	2.60	3.33	0.52	48
**n=9**	387	42	101	18	1.84	1.1	3.99	29	434	1.86	18.8	1.30	6.9	3821	32025	6.21	17.7	3.02	108
C. cyatophora	62	4.32	181	1.73	0.04	0.03	0.22	1.64	161	0.72	2.15	0.19	0.43	3296	14486	3.94	1.17	0.29	15.2
**n=7**	175	13	279	3.22	0.25	0.16	1.65	5.14	219	0.92	6.83	0.70	1.4	4841	21272	4.68	5.48	0.90	39
D. erythraenus	22	17.8	14.6	0.43	0.25	0.54	0.19	4.60	127	0.20	5.76	1.17	18.4	3318	10800	7.88	0.51	0.17	27
**n=9**	123	57	41	2.9	0.4	2.05	1.92	15.7	222	1.03	11.4	2.14	37	5700	18762	12.6	1.3	0.6	83
***Haliclona* sp.1**	21	2.30	26	1.63	1.28	0.10	0.09	5.15	150	0.15	1.23	0.10	1.20	2391	13663	3.66	0.72	0.40	65
**n=9**	171	18	79	10.5	3.25	0.7	0.6	13.5	834	2.05	10.8	1.93	6.2	4972	26315	9	4.97	0.92	249
***Haliclona* sp.2**	99	8.28	21	2.22	0.70	0.32	0.72	24.5	244	0.50	3.96	0.10	2.39	1544	14197	3.49	5.30	0.80	109
**n=4**	128	19	212	3.85	0.87	0.97	0.84	33	435	2.25	9.3	1.56	4.7	2517	19491	4.9	8.9	1.4	271
H. arabica	263	6.38	114	2.98	0.07	0.14	0.73	10.8	249	0.93	8.71	0.08	0.73	3700	11820	2.39	3.55	0.72	25
**n=5**	712	24	145	10.8	0.41	0.96	4.52	25	2906	3.68	64	0.41	2.3	5453	17146	5.17	32	1.90	57
H. erecta	694	13.8	34	9.42	0.24	0.89	2.77	12.4	1413	1.56	44.8	2.60	6.68	1696	12382	5.56	56	4.63	219
**n=3**	2538	17	51	19.9	0.59	1.97	5.34	39	1763	2.87	69	3.30	14.2	2770	16212	9.7	114	6.4	385
N. magnifica	26	6.30	24	1.04	1.14	0.07	0.08	3.07	89	0.50	2.54	0.13	0.43	3460	10678	4.20	1.02	0.13	28
**n=6**	87	68	47	5.63	2.25	0.13	1.26	10.2	172	0.77	3.83	0.24	2.1	5134	15923	8.68	3.21	0.49	56
N. rowi	45	8.41	25	0.88	0.07	0.55	0.24	7.63	219	0.33	2.56	0.14	10.6	556	6537	0.17	3.03	0.22	32
**n=14**	284	73	93	4.5	0.7	8.5	2.36	26	811	2.04	7	0.60	112	3351	25413	3.1	18.5	6.72	441
S. siphonella	43	11.9	47	1.50	0.36	0.16	0.60	7.20	273	1.50	4.50	0.35	1.40	3029	21984	1.14	3.01	0.69	67
**n=8**	531	24	205	6.81	2.17	0.40	6.39	47	768	4.13	10.8	1.04	7.8	6013	52986	2.88	15	1.45	216
S. carteri	63	2.10	16.1	1.60	0.10	0.10	0.10	25.7	219	0.30	2.10	0.10	0.98	1724	12512	3.74	1.30	0.60	45
**n=4**	122	2.68	27	4.73	0.3	0.3	0.4	35	258	0.60	2.7	0.40	3.5	2480	15185	6.8	3.97	0.8	67
S. clavatus	18	0.95	9.21	0.17	2.79	11	0.08	1.54	810	0.22	4.54	0.01	267	440	3378	N.D	0.74	0.20	563
**n=7**	198	2.76	28	2.07	7.57	41	1.33	3.81	1418	0.57	12.2	0.02	782	765	9109		4.63	1.38	1147
T. swinhoei	92	2572	36	126	0.09	0.74	0.30	5.44	224	0.19	6.38	1.11	11.3	2301	5165	15.9	6.09	0.62	15.8
**n=7**	783	19646	89	17169	0.59	2.11	0.96	12.5	442	1.41	17.4	3.37	26	6522	15036	38	21	1.75	55
T. aqabaensis	57	0.82	6.62	1.04	0.04	0.47	0.88	3.36	183	0.42	2.82	N.D	1.64	941	2955	N.D	3.99	0.81	9.6
**n=8**	220	13.8	22	3.75	0.19	4.4	2.55	8.32	848	1.21	9.3		3.9	1534	11686		24	2.72	26
